# Isolation of Rhizosphere Bacteria That Improve Quality and Water Stress Tolerance in Greenhouse Ornamentals

**DOI:** 10.3389/fpls.2020.00826

**Published:** 2020-06-16

**Authors:** Nathan P. Nordstedt, Michelle L. Jones

**Affiliations:** Department of Horticulture and Crop Science, Ohio Agricultural Research and Development Center, The Ohio State University, Wooster, OH, United States

**Keywords:** drought, floriculture, horticulture, photosynthesis, plant–microbe interaction, plant growth promoting rhizobacteria, post-production

## Abstract

Water deficit stress is a major contributor to the loss of ornamental crop value due to its negative effects on plant growth and flowering. In addition, post-production water stress can reduce the photosynthetic capacity of plants, negatively impacting crop quality at retail and in the consumer’s home and garden. While the application of microbe-containing biostimulant products can increase stress tolerance and crop quality, the success of most commercially available biostimulants in greenhouse production systems is inconsistent. To identify beneficial bacteria with consistent biostimulant activity in greenhouse ornamentals, our study isolated bacteria from the rhizosphere of water stressed greenhouse ornamentals. Five species of popular ornamentals were obtained from 15 different commercial greenhouse facilities and then subjected to three cycles of water stress. Over 1,000 bacterial isolates were collected from the rhizosphere and screened *in vitro* for osmoadaptability and 1-aminocyclopropane-1-carboxylate (ACC) deaminase activity. Eighty selected isolates were evaluated in a high-throughput greenhouse trial for their ability to increase plant size and flower number of water-stressed *Petunia* × *hybrida*. Ten bacterial strains selected from the high-throughput trial were then evaluated in a greenhouse validation trial for their ability to increase plant growth and to mitigate the reduction in photosynthetic parameters of water stressed *P. hybrida* and *Pelargonium* × *hortorum*. Application of certain bacteria increased plant size in both species and flower number of *P. hybrida* after recovery from water stress when compared to untreated water stressed plants. In addition, bacteria application increased the chlorophyll fluorescence parameters including quantum yield and efficiency of photosystem II (PSII) and electron transport rate (ETR), while decreasing the extent of electrolyte leakage during water stress and recovery. Overall, this study identified bacterial strains that can increase tolerance to and recovery from water stress in two commercially important ornamental crop species.

## Introduction

Water stress during ornamental plant production impacts crop quality by reducing plant size and flower number, and increasing leaf yellowing and plant wilting (Sánchez-Blanco et al., 2009). These impacts are particularly detrimental to ornamental crops because their value is largely dependent on their appearance. In addition, water stress can have a long-term negative impact on photosynthetic parameters and plant health (Caser et al., 2017). Maintaining optimal photosynthetic efficiency, yield, and electron transport rate (ETR) are essential for protecting plants from photoinhibition during abiotic stress (Singh and Raja Reddy, 2011), and water deficits can lead to decreases in these parameters even before visible symptoms occur (Galmés et al., 2007; Naumann et al., 2007; Singh and Raja Reddy, 2011; Huang et al., 2013).

Plants unable to maintain health and quality under stressful conditions are likely to become unsalable at retail. It has been estimated that 20–30% of ornamental plants either become unmarketable or must be sold at a reduced price because of exposure to environmental extremes (i.e., abiotic stress) or poor post-production care and handling (Nelson and Carlson, 1987; Armitage, 1993; Healy, 2009). In addition to the more obvious impacts on visual qualities, abiotic stress can also permanently affect photosynthesis, such that plants are unable to fully recover and regain photosynthetic capacity equivalent to pre-stress levels (Armitage et al., 1983). Plants that have reduced photosynthetic efficiency may not grow as well in the consumer’s home or garden, and their ability to survive under subsequent exposure to stresses will be reduced (Siddique et al., 2016). Therefore, growers need additional tools to make ornamental plants more tolerant of abiotic stresses and reduce crop losses.

A potential tool for growers to increase plant tolerance to abiotic stress is the application of plant growth promoting rhizobacteria (PGPR), a class of microorganisms that can colonize plant roots and produce enzymes and secondary metabolites that positively influence plant stress tolerance. Noteable PGPR include many genera such as *Acinetobacter*, *Agrobacterium*, *Arthobacter*, *Azospirillum*, *Bacillus*, *Bradyrhizobium*, *Burkholderia*, *Pseudomonas*, *Rhizobium*, and *Serratia* (Goswami et al., 2016). In particular, the application of PGPR in ornamental plant production can be used to enhance plant stress tolerance and increase plant health and quality under water deficient conditions. Recent studies have shown that the application of different PGPR can enhance plant quality by increasing plant size and flower number in greenhouse ornamental species when grown under normal conditions or subjected to abiotic stress (Göre and Altin, 2006; Flores et al., 2007; Hoda and Mona, 2014; Leoni et al., 2019; Nordstedt et al., 2020). However, to positively influence the quality of plants subjected to abiotic stress, the PGPR must first induce physiological changes that increase plant stress tolerance, allowing the plant to better overcome the stress during recovery (Yang et al., 2009).

Increased plant stress tolerance can be accomplished through a variety of mechanisms, many of which are likely interconnected. A key mechanism for PGPR to induce stress tolerance in plants is to modify hormone levels, resulting in enhanced growth. PGPR can reduce stress-induced ethylene in plants via the production of the enzyme 1-aminocyclopropane-1-carboxylate (ACC) deaminase, which acts by cleaving ACC, the direct precursor to ethylene (Klee et al., 1991). Through the activity of ACC deaminase, stress ethylene remains below inhibitory levels, allowing plants to maintain normal root growth and contributing to delayed senescence under water stress (Glick et al., 2007). The onset of water stress can also reduce the endogenous levels of auxin produced in plants (Nilsen and Orcutte, 1996), which can be partially mediated by bacterial auxin production (Ngumbi and Kloepper, 2016). Elevated levels of abscisic acid (ABA) modulate plant physiological processes during water stress such as stomatal closure and reduction in carboxylation, which temporarily saves resources by reducing energy production (Farooq et al., 2009). Application of PGPR can offset this energy loss by stimulating photosynthesis while also increasing ABA content and water use efficiency in water stressed arabidopsis (Bresson et al., 2013; Cohen et al., 2015).

In addition to regulating upstream processes such as phytohormone production, stomatal closure, and carboxylation levels, PGPR can also positively influence parameters such as membrane integrity and the quantum efficiency of photosystem II (PSII) in plants during water stress and recovery. PGPR reduce electrolyte leakage in water stressed plants by increasing membrane integrity, which leads to improved recovery after stress (Vardharajula et al., 2011; Jodeh et al., 2015; Tiwari et al., 2016). Measuring the quantum efficiency of PSII in plants under severe water stress can serve as an indicator of plant health during stress and recovery. Application of *Bacillus* spp. to potato and *Burkholderia phytofirmans* and *Enterobacter* sp. to maize significantly increased the quantum efficiency of PSII in these plants under severe water stress (Gururani et al., 2013; Naveed et al., 2014).

As resources like water become increasingly limited, all of agriculture, including the greenhouse industry, will need to adopt solutions that will allow them to continue producing high quality crops with fewer inputs. The application of PGPR in the form of commercial products (biostimulants) is a promising tool to produce more resilient crops with the ability to withstand severe water stress while maintaining health and quality during recovery. Unfortunately, most biostimulant research has focused on field crops, and the efficacy of these products in greenhouse production can be highly variable (Paulitz and Richard, 2001; Ngumbi and Kloepper, 2016). Noteable differences in greenhouse production compared to field production are the use of soilless media with less biodiversity, production in containerized systems, and increased frequency of fertilizer application (Montagne et al., 2017). Microbial inoculants have been isolated from a variety of natural environments, most of which are drastically different than controlled environment greenhouse production systems. The surrounding environment dramatically influences the root-associated bacterial communities, and results from other trials may not translate into practical applications if cultural inputs are not similar (Ngumbi and Kloepper, 2016).

Studies have shown that isolating bacteria from environments that are similar to ones they will be used in is an effective strategy for identifying PGPR strains for a particular cropping system (Zahir et al., 2008; Ali et al., 2013). In particular, the isolation of bacteria directly from the rhizosphere of plants subjected to water deficit conditions can identify PGPR with the ability to confer water stress tolerance when applied to other plants (Liu et al., 2013; Wang et al., 2014; Huang et al., 2017; Yasmin et al., 2017; Raheem et al., 2018). Therefore, the aim of this study was to establish a novel collection of bacteria for the purpose of identifying strains that improve ornamental plant tolerance to water stress. The bacteria were isolated from the rhizosphere of plants grown in controlled environment greenhouse systems and subjected to severe water deficit. The isolated bacteria were screened using a previously established pipeline (Nordstedt et al., 2020) and then evaluated for their ability to improve the growth, quality, and overall health of two economically important ornamental plant species.

## Materials and Methods

### Isolation of Rhizospheric Bacteria From Greenhouse-Grown Ornamental Plants

*Solenostemon scutellarioides* (coleus), *Petunia* × *hybrida* (petunia), *Pelargonium* × *hortorum* (geranium), *Catharanthus roseus* (vinca), and *Zinnia elegans* (zinnia) plants at the mature flowering stage, which had been grown alongside other plants throughout their production cycle, were collected from 15 greenhouse ornamental production facilities in Ohio and West Virginia. These greenhouse facilities were selected for their diversity in environmental and cultural practices (i.e., water source, substrate composition, and chemical inputs). Irrigation water sources between greenhouse facilities varied between municipal/city, well, and recirculated pond water. Fertilizer rates and timing varied between greenhouses; however, each facility fertilized with at least 100 mg L^–1^ N at each fertilization. The soilless growing media components were vastly different between growers and consisted of a variety of formulations using peat, perlite, rice hulls, pine bark, and wood fiber. Plants were brought to the Ohio Agricultural Research and Development Center (Wooster, OH, United States) research greenhouses and subjected to water stress by removing all irrigation until plants were visibly wilted, and then rewatering so that plants regained turgidity. This cycle of wilting and recovery was repeated three times. The stress was repeated to induce plant stress signaling responses and changes to the rhizospheric environment. These changes can alter the microbial population in the rhizosphere, providing a higher likelihood of selecting strains with the ability to withstand water stress (Naylor and Coleman-Derr, 2018).

Following the third water stress cycle, excess potting media was removed from the root system and a 5-g root sample was collected. Samples were vortexed for 1 min in 25 mL sterile H_2_O and then sonicated at 117 V for 1 min (Branson 2200 Ultrasonic Cleaner, Shelton, CT, United States). A 1-mL aliquot of this sample was serial diluted in sterile H_2_O and 100 μL of 10^–2^, 10^–3^, and 10^–4^ diluted solutions were plated onto 1X King’s Media B (KMB) agar plates supplemented with 100 μg/mL cycloheximide to prevent fungal growth. In addition, a 1-mL aliquot of each sample was added to 4 mL 0.1X Tryptic Soy Broth (TSB) and incubated at 65°C for 30 min to select for spore-forming bacteria, while preventing growth of film-producing bacteria such as *Pseudomonas* sp. (Fall et al., 2004; Gutierrez, 2004). The heat-treated samples were serial diluted in sterile H_2_O and 100 μL of 10^–2^, 10^–3^, and 10^–4^ diluted solutions were plated onto 0.1X TSB agar plates supplemented with 100 μg/mL cycloheximide. Agar plates were incubated at 28°C for 48 h followed by 48 h at 4°C to allow for any color development between different isolates. Following incubation, bacterial isolates of unique morphology were struck into single cultures and then stored in 25% glycerol stocks at −80°C. This collection is referred to as the greenhouse rhizosphere collection.

### *In vitro* Selection of Osmoadaptive Bacteria

An *in vitro* osmoadaptability bioassay was conducted according to Nordstedt et al. (2020) for the selection of the most osmotic stress tolerant bacteria within the greenhouse rhizosphere collection. Briefly, a sterile 96-well microplate replicator (Boekel Scientific, Feasterville, PA, United States, Model 140500) was used to transfer glycerol freezer stocks of each isolate into separate wells of a 96-well microtiter plate pre-filled with 200 μL Luria-Bertani (LB) media for inoculation. Bacteria were incubated at 28°C with shaking at 120 r/min for 18 h. The optical density at 595 nm (OD_595_) was measured using a spectrophotometer (DTX880, Beckman Coulter, Brea, CA, United States) and adjusted to OD_595_ of 0.8 with LB. Ten microliters of the bacteria cultures were transferred to a microtiter plate prefilled with yeast extract mannitol (YEM) broth amended with 30% PEG_8000_ (w/v). Each bacterial isolate was assayed on three separate microtiter plates (*n* = 3). Strains *Pseudomonas poae* 29G9 and *Pseudomonas fluorescens* 90F12-2 were used for comparison, because they were previously reported to withstand osmotic stress and increase plant tolerance to abiotic stress (Nordstedt et al., 2020). Plates were incubated at 28°C with shaking at 120 r/min. After 96 h, the OD_595_ was measured to quantify the growth of the bacteria under osmotic stress.

### Selection of ACC Deaminase-Producing Bacteria

To identify ACC deaminase-producing bacteria, bacteria were first grown in media containing ACC as the sole nitrogen source. A sterile 96-well microplate replicator was used to transfer freezer glycerol stocks of each isolate to individual wells in a 96-well microtiter plate containing 200 μL of amended Dworkin and Foster (DF) minimal media (Dworkin and Foster, 1958). For a liter of DF minimal media, the following was added to water: 4.0 g of KH_2_PO_4_, 6.0 g of Na_2_HPO_4_, 0.2 g of MgSO_4_.7H_2_0, 2.0 g of glucose, 2.0 g of gluconic acid, 2.0 g of citric acid, 1.0 mg of FeSO_4_.7H_2_0, 10 μg of H_3_BO_3_, 10 μg of MnSO_4_, 70 μg of ZnSO_4_, 50 μg of CuSO_4_, and 10 μg of MoO_3_; supplemented with 3 mM ACC as the sole source of nitrogen. Bacterial isolates were incubated at 28°C for 48 h shaking at 120 r/min. The OD_595_ was measured using a spectrophotometer (DTX880, Beckman Coulter) to select bacteria able to grow in the DF-ACC media. ACC deaminase activity was then quantified for each bacterial isolate able to grow in the media.

ACC deaminase activity from the selected isolates was quantified using a colorimetric enzyme activity assay adapted from Penrose and Glick (2003) and optimized for the high-throughput evaluation in a 96-well plate format. *Pseudomonas putida* UW4^+^ was used as the positive bacteria control because it has been well studied as an ACC deaminase producer with beneficial effects on plant stress tolerance (Hontzeas et al., 2004). Bacteria were cultured in 12 mL TSB divided between four culture tubes and incubated at 28°C for 48 h shaking at 200 r/min. Cultures were combined into two tubes and bacteria were harvested by centrifugation at 4,000 *g* for 10 min at 4°C. The cells were washed with 5 mL DF media and the centrifugation was repeated. Cells were resuspended in 3 mL DF media supplemented with ACC at a final concentration of 3 mM and incubated at 28°C for 24 h shaking at 200 r/min. The cultures were then combined into a single tube and harvested by centrifugation at 4,000 *g* for 10 min at 4°C. The supernatant was decanted, and the cells were washed three times in 6 mL of 0.1 M Tris-HCl (pH 7.6) and resuspended in 1.8 mL of 0.1 M Tris-HCl (pH 8.5). Toluene (90 μL) was added to each tube and samples were vortexed three times for 1 min and then stored on ice. A 190 μL aliquot of the toluenized cells was added to each of three wells (*n* = 3) in a 96-well 2 mL plate containing 19 μL of 0.5 M ACC. Two wells in each plate contained 19 μL distilled H_2_O to serve as the negative control. Samples were incubated in a 30°C water bath for 15 min. Following the addition of 950 μL 0.56 M HCl to each well, 800 μL of each sample was transferred to a 1.7 mL centrifuge tube, and samples were centrifuged at 13,000 *g* for 5 min. Three-hundred microliters of the supernatant was mixed with 240 μL of 0.56 HCl and 90 μL of 2,4-dinitrophenylhydrazine reagent (0.2% 2,4-dinitrophenylhydrazine in 2 M HCl), and samples were incubated in a 30°C water bath for 30 min. Following incubation, 600 μL of 2 M NaOH was added to each sample and the absorbance of the mixture was measured at 540 nm (A_540_) using a spectrophotometer (DTX880, Beckman Coulter). An increase in absorbance served as an indicator of α-ketobutyrate production, and confirmation of the cleavage of ACC by ACC deaminase.

### High-Throughput Greenhouse Evaluation of *in vitro*-Selected Bacterial Isolates

A high-throughput greenhouse trial pipeline developed by Nordstedt et al. (2020) was used for the *in planta* evaluation of the *in vitro*-selected osmoadaptive and ACC deaminase-producing bacteria. The bacteria were evaluated for their ability to stimulate growth of *Petunia* × *hybrida* ‘Picobella Blue’ under water stress conditions. Seeds were sown in Pro-Mix PGX media (Premier Tech Horticulture, Quakertown, PA, United States) and fertilized at each irrigation with 50 mg L^–1^ N from 15N–2.2P–12.5K–2.9Ca–1.2Mg water soluble fertilizer (JR Peters Inc., Allentown, PA, United States). Seedlings were transplanted 3 weeks after sowing to 6.4 cm pots containing Pro-Mix PGX media. Plants were arranged in a randomized complete block design (RCBD) with six blocks, and two replicates per block (*n* = 12). Due to the large number of bacterial isolates to be tested, three independent greenhouse trials were conducted, and results were analyzed separately. The greenhouse was equipped with supplementary lighting powered by high-pressure sodium and metal halide lamps (GLX/GLS e-systems GROW lights, PARSource, Petaluma, CA, United States) for a 16-h photoperiod. During the light period, the supplementary lighting system was turned on when the PAR flux was below 250 μmol m^–2^ s^–1^ at bench level and turned off when the PAR flux reached 350 μmol m^–2^ s^–1^. Air temperature at canopy height was maintained at 25/19 [standard deviation (SD) ± 3]°C day/night.

Bacterial inoculum was prepared by inoculating LB media with individual bacteria cultures (selected from the *in vitro* osmoadaptability and ACC deaminase activity assays) and incubating at 28°C for 16 h with shaking at 250 r/min. Cultures were then adjusted to an OD_595_ = 0.8 with LB. Inoculum for treating plants was prepared by diluting each culture 1:100 in reverse osmosis (RO) water. Uninoculated LB diluted 1:100 with RO water was used as the negative control. Plants were treated weekly with a media drench of 40 mL bacterial inoculum.

Water stress was induced 3 weeks post-transplant by discontinuing irrigation and bacteria treatments until all plants in the experiment showed a visible loss of turgidity across the plant. Plants were then rewatered and bacteria treatments were resumed. Plant performance of previously water stressed plants was evaluated 6 weeks post-transplant. Flower numbers (open flowers and buds showing color) and shoot (stems and leaves) biomass were used as indicators of plant performance. Shoot tissue was dried at 49°C for at least 96 h and then weighed to measure dry biomass.

### Sequencing and Taxonomic Classification of Selected Bacteria

Genomic DNA was extracted from bacteria selected in the high-throughput greenhouse trial using the Quick-DNA Bacterial Miniprep kit (Zymo Research, Irvine, CA, United States) and samples were submitted to CoreBiome (St. Paul, MN, United States) for whole-genome sequencing. Samples were quantified with Qiant-iT Picogreen dsDNA Assay (Invitrogen, Carlsbad, CA, United States). Libraries were prepared with a procedure adapted from the Nextera Library Prep kit (Illumina, San Diego, CA, United States). Libraries were sequenced on an Illumina NovaSeq using singled-end 1 × 100 reads with a NovaSeq SP flowcell. DNA sequences were filtered for low quality (Q-Score < 20) and length (<50), and adapter sequences were trimmed using cutadapt (v.1.15). The sequences for each sample were assembled into contigs using SPAdes (v3.11.0). Contigs greater than 1,000 bases in length were used in a QUAST (QUAST v4.5) analysis. Prokka (v 1.12) was used to annotate genes for each strain using the contigs > 1,000 bases as described above. Sequence files were uploaded to the Microbial Genome Atlas (MiGA) for taxonomic classification. MiGA allows for the computing of average nucleotide identity (ANI) between the query sequence and the NCBI prokaryotic genome database (Rodriguez-R et al., 2018). Query sequences sharing greater than 94% ANI with a species in the database were considered the same species. Bacteria that did not share at least 94% ANI with another sequence were considered to not have a species-level match in the database and were given the “species” designation (i.e., *Pseudomonas* sp.) (Konstantinidis and Tiedje, 2005).

### Data Deposition

The whole-genome sequence data were deposited in the NCBI database under the BioProject no PRJNA631210.

### Evaluation of Quality and Stress Tolerance of Water Stressed Ornamental Plants Treated With Bacteria

#### Greenhouse Validation Trial

A greenhouse validation trial was used to evaluate the effect of applying individual bacteria selected from the high throughput trial (Table 1) on the quality and water stress tolerance of *P. hybrida* (petunia) and *Pelargonium* × *hortorum* (geranium) plants. *P. putida* UW4^+^ was included because it is well-documented to increase quality and stress tolerance of plants subjected to abiotic stress (Hontzeas et al., 2004). The negative control plants were treated with uninoculated LB and subjected to water stress. The positive control plants were also treated with uninoculated LB but were continually irrigated and not subjected to water stress. *P. hybrida* ‘Picobella Blue’ and *P. hortorum* ‘Maverick Red’ seeds were sown and cultivated as described for the high-throughput trial, with the exception that seedlings were transplanted to 11.4 cm pots to simulate common production practices. Greenhouse conditions were maintained similar to the high-throughput trial. Plants grown for the evaluation of quality were arranged in a RCBD with 16 blocks and one replicate per block (*n* = 16). Additional plants were grown in a RCBD to evaluate plant stress tolerance with six blocks and one replicate per block (*n* = 6).

**TABLE 1:** The 10 bacterial strains selected from the high-throughput greenhouse trial for their ability to increase flower number or plant biomass of water stressed *Petunia* × *hybrida* ‘Picobella Blue.’

			*In vitro* selection		
Strain	Host plant isolated from	Heat- treated isolation	YEM + PEG	ACC deaminase activity	Taxonomic classification
C9C5	Coleus	X	X		*Arthrobacter* sp.
C4D7	Geranium	X	X		*Pseudarthrobacter* sp.
C5G2	Coleus		X		*Leifsonia* sp.
C2B4	Zinnia			X	*Pseudomonas* sp.
C6C2	Coleus			X	*Herbaspirillum robiniae*
C2F7	Petunia			X	*Pseudomonas brassicacearum*
C7D2	Zinnia			X	*Pseudomonas corrugata*
C9C3	Coleus			X	*Herbaspirillum* sp.
C1C7	Geranium			X	*Pseudomonas* sp.
C8A5	Coleus			X	*Pseudomonas corrugata*

*These bacteria were isolated from the rhizosphere of greenhouse ornamental plants and previously selected for their ability to grow under PEG-mediated osmotic stress (YEM + PEG) or activity of ACC deaminase in vitro.*

Bacterial inoculum was prepared similar to the high-throughput trial, and the plants were treated weekly with 120 mL diluted inoculum to account for the larger pot size and media volume. Water stress began 5 weeks after transplant by discontinuing all irrigation and bacteria treatments. Water stress was induced for 7 and 10 days for petunia and geranium, respectively, to the point of loss of turgidity across the plant. Plants were rewatered and weekly bacteria treatments were resumed for 3 weeks, at which point all plants were at the final marketable stage (i.e., flowering).

#### Plant Growth and Quality

Flower number and plant biomass were measured as indicators of plant growth and quality (*n* = 16). When plants reached the final marketable stage, flower number (open flowers and buds showing color) was recorded, and flowers and shoots (stems and leaves) were individually harvested, dried at 49°C for at least 96 h, and weighed to measure dry biomass.

#### Plant Stress Tolerance

Electron transport rate, quantum yield, and quantum efficiency of PSII were measured to evaluate differences in photosynthetic health involved in plant stress tolerance (*n* = 6). ETR, quantum yield, and quantum efficiency of PSII were measured using an open gas-exchange system (Li-6400 XT; Li-Cor Inc., NE, USA, United States) with an integrated fluorescence chamber head (Li-6400-40 leaf chamber fluorometer; Li-Cor Inc., Lincoln, NE, United States). A lower, fully expanded leaf was used, and all measurements were taken the day prior to water stress (T_1_), at severe water stress (T_2_), and 3 days after rewatering to assess recovery from water stress (T_3_). To identify bacterial strains that improved the photosynthetic health of plants during recovery, the mean of each treatment was ranked for each measurement, and the individual ranking values were summed together to provide an overall ranking (Table 2). Electrolyte leakage was measured from plants at T_1_ and T_3_ by collecting three 6-mm leaf punches from the same leaf used for photosynthetic measurements.

**TABLE 2:** Influence of bacterial application on the photosynthetic health of *Petunia* × *hybrida* ‘Picobella Blue’ plants under severe water stress (T_2_) and following recovery (T_3_).

Severe water stress (T_2_)					Recovery (T_3_)				
Strain	ETR	Fv’/Fm’	ΦPSII	Rank	Strain	ETR	Fv’/Fm’	ΦPSII	Rank
C7D2	44.07	0.75	0.67	1	C7D2	43.23	0.751	0.659	1
C1C7	43.44	0.738	0.66	2	C9C3	42.93	0.743	0.653	2
C2B4	43.25	0.737	0.656	3	C2B4	42.92	0.742	0.653	3
C2F7	41.21	0.706	0.625	4	C9C5	42.86	0.744	0.653	4
C8A5	40.64	0.699	0.617	5	C1C7	42.91	0.741	0.653	5
C4D7	40.37	0.694	0.614	6	C6C2	42.62	0.747	0.649	6
C5G2	39.48	0.684	0.599	7	C5G2	42.4	0.741	0.646	7
UW4 +	38.53	0.671	0.585	8	C4D7	42.15	0.729	0.642	8
C9C3	38.14	0.664	0.58	9	UW4 +	42.1	0.735	0.641	9
(−) Control	38.15	0.626	0.579	10	C8A5	41.79	0.728	0.636	10
C9C5	35.6	0.628	0.54	11	C2F7	40.97	0.721	0.623	11
C6C2	33.18	0.609	0.504	12	(−) Control	40.79	0.694	0.621	12
Mean	39.67	0.684	0.602		Mean	42.31	0.735	0.644	
SD	3.23	0.046	0.049		SD	0.79	0.016	0.012	

*Electron transport rate (ETR), efficiency of photosystem II (Fv’/Fm’), and quantum yield of photosystem II (ΦPSII) were measured on a single leaf of each plant per treatment (n = 6) and the mean value for each is shown. Plants treated with strains from the greenhouse rhizosphere collection were compared to the bacterial treatment control strain UW4^+^ and the uninoculated negative control. Measurements were taken 7 days after withholding water for water stress (T_2_) and 3 days after rewatering for recovery (T_3_), respectively. Means are organized based on if they are more than one standard deviation (SD) greater than the overall mean (light gray), greater than the mean but less than one SD (gray), lower than the mean but not by more than one SD (dark gray), and more than one SD lower than the mean (black). The mean values for each photosynthetic measurement were ranked from highest to lowest value, and the individual ranks were summed together to get the overall rank. Treatments with the lowest ranking sum were given the best overall rank.*

#### Electron Transport Rate, Quantum Yield, and Efficiency of Photosystem II

Chlorophyll fluorescence measurements were taken between 10:00 and 12:00. The instrument conditions were set as follows: 25°C cuvette temperature, 150 μmol photon m^–2^ s^–1^ to ensure light saturation, CO_2_ concentration of 400 μmol CO_2_ mol^–1^, air and leaf vapor pressure deficit between 2 and 2.5 kPa. After inducing photosynthesis under the above conditions and once steady state was reached, the following parameters were measured of light-adapted leaves: ETR, PSII efficiency (Fv’/Fm’), and quantum yield of PSII (φPSII).

Electron transport rate was calculated by assuming a leaf absorption of 0.85 and a PSII:PSI ratio of 1:1 (ETR = PPFD × φPSII × 0.85 × 0.5). The efficiency of PSII was calculated as: Fv’/Fm’ = (Fm’ – Fo’)/Fm’, where Fo’ is the minimal fluorescence in the light-adapted state and Fm’ is the maximal value. Relative quantum yield of PSII (φPSII) was calculated as: φ_*PSII*_ = (Fm’ - Fs)/Fm’, where Fs is the steady state parameter (Huang et al., 2013).

#### Electrolyte Leakage

Leaf tissue was stored in a 15 mL plastic tube on ice during collection. Tissue was triple rinsed with 1 mL nanopure H_2_O and then stored in 4 mL nanopure H_2_O. Tubes were gently agitated with shaking at 120 r/min for 2 h and electrolyte content (EL_1_) was measured using the Accumet AB30 Conductivity Meter (Fisher Scientific, Oslo, Norway). Tissue was then autoclaved for 20 min and final electrolyte content (EL_2_) was measured. Total electrolyte leakage percentage was defined as: (EL%) = (EL_1_/EL_2_) × 100.

### Statistical Analysis

Results from each of the three high-throughput greenhouse trials were analyzed independently. Data for each phenotype (flower number and shoot dry weight) were analyzed using a linear mixed effect model with lme4 (Bates et al., 2015), and fixed effect estimates (FEE) for treatment effect were used to rank each treatment best to worst compared to the negative control using the highest to lowest FEE, respectively. Rankings of both phenotypes (flower number and shoot biomass) were summed and these values were again ranked best to worst. Treatments ranking in the top 10% for each individual greenhouse trial were selected for further evaluation.

For the greenhouse validation trial, statistical analyses were conducted in R Studio version 3.5.2 using an analysis of variance (ANOVA) with the model: Y = μ + treatment + block. Factors that had a significant *p*-value (*p* < 0.05) were analyzed using Dunnett’s test with the negative control used for comparison.

## Results

### Isolation of the Greenhouse Rhizosphere Collection

A total of 1,056 bacterial isolates were collected from the rhizosphere of greenhouse-produced coleus, petunia, geranium, vinca, and zinnia plants. Of the total isolates, 609 were selected on KMB and 447 were selected on TSB following heat treatment.

### *In vitro* Selection of Osmoadaptive and ACC Deaminase Producing Bacteria

**FIGURE 1:**
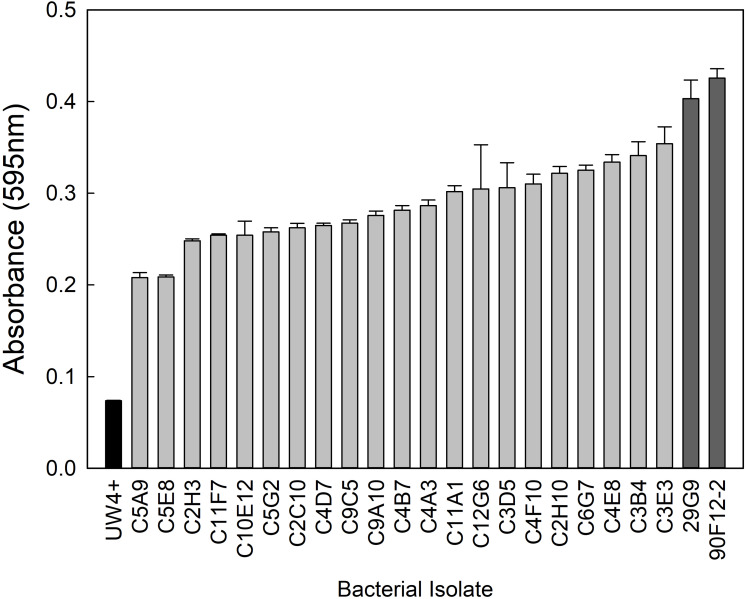
Growth of bacterial isolates in YEM media containing 30% polyethylene glycol to induce osmotic stress. Bars represent the mean (±SE) optical density (OD_595_) of the isolates after 96 h incubation at 28°C (*n* = 3). Isolates with an absorbance greater than 0.2 (light gray) were selected for further evaluation. Strains 29G9 and 90F12-2 (dark gray) were used for comparison because they were previously reported to increase plant tolerance to abiotic stress.

**FIGURE 2:**
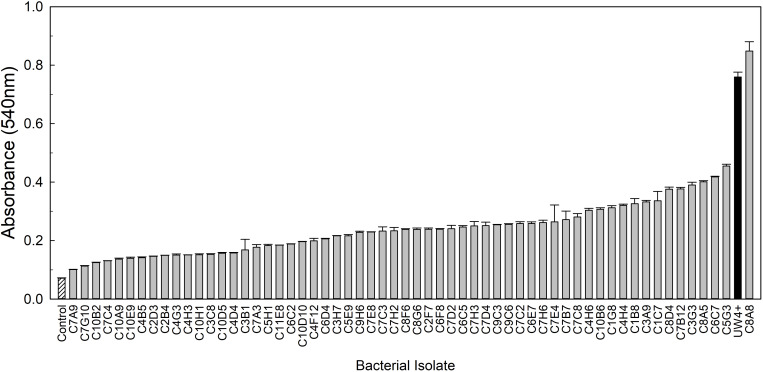
ACC deaminase activity of bacterial isolates measured by a 2,4-dinitrophenylhydrazine assay after induction in a Dworkin and Foster (DF) media containing ACC as the sole nitrogen source. Bars represent the mean (±SE) absorbance (A_540_) of the ACC byproduct, α-ketobutyrate, following the enzymatic reaction (*n* = 3). Bacterial cultures (light gray) were compared to the uninoculated control (white with lines) and the positive bacteria control *Pseudomonas putida* UW4^+^ (black).

From the greenhouse rhizosphere collection, a total of 80 isolates were selected from the *in vitro* bioassays. Of these isolates, 21 had an OD_595_ greater than 0.2 and were identified as the most osmoadaptive, although the growth of each isolate was less than the comparison strains 29G9 and 90F12-2 (Figure 1). The greenhouse rhizosphere collection was also tested for ACC deaminase activity, and 59 isolates tested positive for enzyme activity (Figure 2). There were no isolates that were selected by both assays (osmoadaptability and ACC deaminase activity).

### High-Throughput Greenhouse Evaluation of *in vitro*-Selected Bacteria

The 80 bacterial isolates selected from the *in vitro* bioassays were evaluated in three independent greenhouse trials for their ability to increase plant size and/or flower number of water-stressed petunia plants when compared to the negative control. Treatments ranking in the top 10% for each individual greenhouse trial were selected for further evaluation (Supplementary Table S1).

From the high-throughput greenhouse trials, 10 isolates were selected for increasing plant size and/or flower number when compared to the negative control: C4D7, C7D2, C1C7, C5G2, C6C2 C8A5, C9C3, C9C5, C2B4, and C2F7. Following taxonomic identification (see below), these strains were evaluated in a validation greenhouse trial under water stress (Table 1).

### Taxonomic Classification

The 10 bacterial isolates selected from the high-throughput greenhouse trial were sequenced and taxonomic classification was assigned (Table 1). These bacteria are from five different genera, with five of them belonging to the genus *Pseudomonas*.

### Shoot Biomass and Flower Number of Water Stressed Plants When Treated With Beneficial Bacteria

**FIGURE 3:**
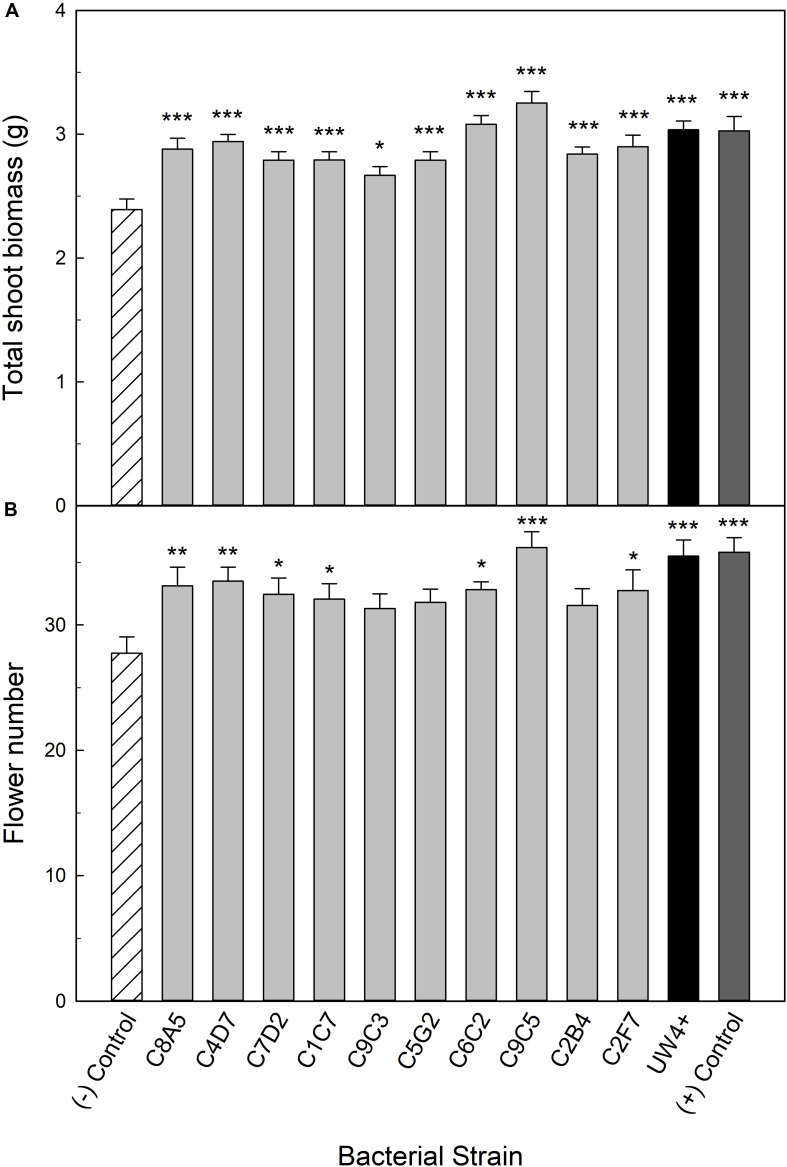
Plant growth performance parameters for *Petunia* × *hybrida* ‘Picobella Blue,’ plants subjected to water stress 5 weeks after transplant (*n* = 16). Plants were treated with bacterial inoculum weekly starting at transplant. Plants treated with strains from the greenhouse rhizosphere collection (light gray), were compared to the bacterial treatment control strain UW4^+^ (black), the uninoculated negative control (white with lines), and the uninoculated positive control (dark gray). The negative control plants were subjected to water stress similar to the inoculated plants, whereas the positive control plants were irrigated regularly throughout the experiment. Total shoot biomass (dry weight) **(A)** and number of flowers **(B)** was measured 3 weeks after rewatering following severe water stress. Bars represent mean (±SE). The asterisks indicate significance between the treatments compared to the uninoculated negative control using the Dunnett’s test; *, **, *** significant at *P* ≤ 0.05, 0.01, or 0.001, respectively.

The 10 bacterial strains selected from the high-throughput trial were evaluated for their ability to increase water stress tolerance in petunia and geranium plants. Application of bacteria had broad beneficial effects on the shoot biomass of petunia plants recovering from water stress. Each bacterial application significantly increased shoot biomass compared to the negative control, an average increase of 21% (Figure 3A). In addition to shoot biomass, seven of the greenhouse rhizosphere strains (C8A5, C4D7, C7D2, C1C7, C6C2, C9C5, C2F7) and UW4^+^ significantly increased flower number, with an average increase of five flowers per plant compared to the negative control (Figure 3B). Treatment with strain C9C5 had the greatest effect on plant quality, increasing shoot biomass by 36% and increasing flower number by at least eight flowers per plant when compared to the negative control. Plants treated with C6C2 and C9C5 had greater shoot biomass than plants treated with UW4^+^ and the positive control that did not undergo water stress. In addition, plants treated with C9C5 also had greater flower number than UW4^+^ and the positive control (Figures 3A,B).

**FIGURE 4:**
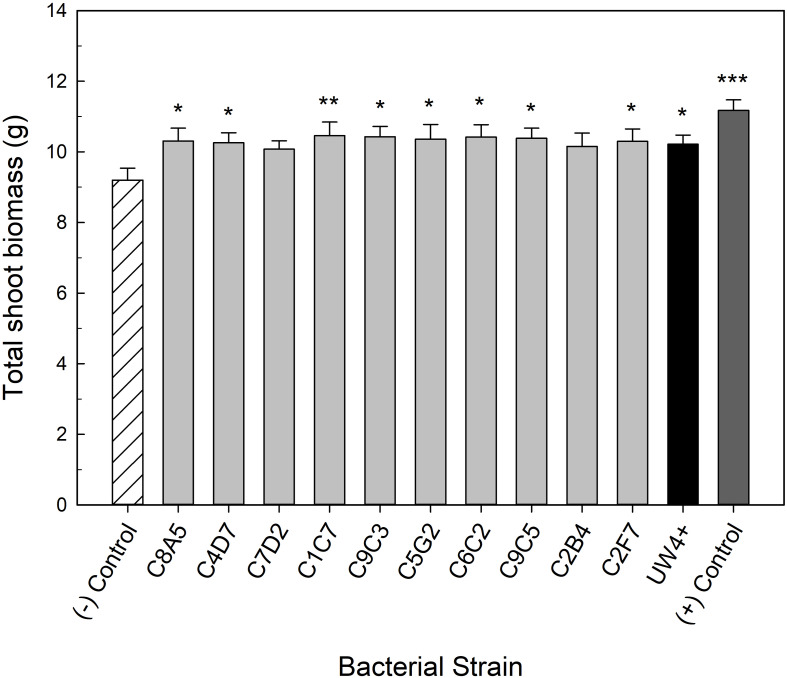
Plant growth performance parameters for *Pelargonium* × *hortorum* ‘Maverick Red,’ plants subjected to water stress 5 weeks after transplant (*n* = 16). Plants were treated with bacterial inoculum weekly starting at transplant. Plants treated with strains from the greenhouse rhizosphere collection (light gray) were compared to the bacterial treatment control strain UW4^+^ (black), the uninoculated negative control (white with lines), and the uninoculated positive control (dark gray). The negative control plants were subjected to water stress similar to the inoculated plants, whereas the positive control plants were irrigated regularly throughout the experiment. Total shoot biomass (dry weight) was measured 3 weeks after rewatering following water stress. Bars represent mean (±SE). The asterisks indicate significance between the treatments compared to the uninoculated negative control using the Dunnett’s test; *, **, *** significant at *P* ≤ 0.05, 0.01, or 0.001, respectively.

Bacteria application also had a beneficial effect on the quality of water stressed geranium. Application of eight of the greenhouse rhizosphere strains (C8A5, C4D7, C1C7, C9C3, C5G2, C6C2, C9C5, C2F7) and UW4^+^ significantly increased shoot biomass when compared to the negative control (Figure 4). The increase in shoot biomass was not as great as the positive control; however, application of the eight strains still ameliorated the negative effects of water stress on plant size when compared to the negative control. There was not an increase in geranium flower number or biomass, likely due to their habit of producing very few but large inflorescences (data not shown).

### Chlorophyll Fluorescence Parameters and Electrolyte Leakage of Bacteria Treated Plants Subjected to Water Stress

#### Electron Transport Rate, Quantum Yield, and Quantum Efficiency of PSII in Petunia

Out of the 11 bacterial strains tested (including UW4^+^), C7D2 was identified as the best strain for increasing overall photosynthetic health of petunia plants. C7D2 increased the ETR, ΦPSII, and Fv’/Fm’ more than one SD greater than the treatment means in petunia throughout severe water stress (T_2_) and recovery (T_3_) (Table 2). Strain C2B4 also showed consistent results in both severe water stress (T_2_) and recovery (T_3_), being ranked in the top three strains under both conditions. Strain C1C7 also increased the photosynthetic health of petunia plants under severe stress (T_2_); however, the effects were not as evident in plants recovering from water stress (T_3_). Plants treated with the control strain UW4^+^ did not have increased photosynthetic health compared to plants treated with most other bacterial strains; however, petunia plants treated with bacteria from the greenhouse rhizosphere collection did tend to rank higher in photosynthetic health than the untreated negative control in terms of ETR, Fv’/Fm’, and ΦPSII (Table 2). Overall, petunia plants treated with bacteria from the collection also recovered from water stress better than the negative control in terms of electrolyte leakage, ETR, Fv’Fm, and ΦPSII. Treated plants had lower percent differences when comparing recovery measurements (T_3_) to measurements prior to water stress (T_1_) (Table 3).

**TABLE 3:** Percent change in physiological measurements of *Petunia* × *hybrida* ‘Picobella Blue’ after 3 days of recovery (T_3_) from being subjected to 7 days of water stress, when compared to measurements prior to water stress (T_1_).

Strain	Electrolyte leakage	ETR	Fv’/Fm’	ΦPSII
(−) Control	54.5	−10.2	−9.5	−9.8
C8A5	7.8	−8.3	−5.0	−8.3
C4D7	10.9	−7.2	−4.7	−6.6
C7D2	10.5	−4.9	−1.4	−3.9
C1C7	16.5	−5.8	−3.1	−5.1
C9C3	17.0	−4.5	−3.5	−4.6
C5G2	13.0	−6.4	−2.9	−5.9
C6C2	–4.9	−6.3	−2.4	−5.8
C9C5	14.1	−5.2	−2.8	−4.7
C2B4	9.3	−6.3	−3.1	−5.4
C2F7	13.5	−9.8	−5.6	−9.5
UW4+	8.8	−7.5	−3.8	−6.6

*Plants treated with strains from the greenhouse rhizosphere collection were compared to the bacterial treatment control strain UW4^+^ and the uninoculated negative control.*

#### Electron Transport Rate, Quantum Yield, and Quantum Efficiency of PSII in Geranium

Unlike the observed results in petunia, the effect of bacteria application on the photosynthetic health (ETR, Fv’/Fm’, and ΦPSII) of geranium was not as consistent throughout water stress and recovery. The strain C6C2 was ranked in the top three strains under severe water stress (T_2_) and recovery (T_3_), being the most consistent treatment to increase the ETR, Fv’/Fm’, and ΦPSII of geranium under both conditions. The control treatment UW4^+^ was the best at increasing overall photosynthetic health of geranium plants under severe water stress (T_2_), as plants treated with UW4^+^ had an increase in ETR, ΦPSII, and Fv’/Fm’ more than one SD greater than the treatment means. Strains C2F7 and C4D7 had the greatest effect on increasing overall photosynthetic health of geranium after recovering from water stress (T_3_). Treatment with each of the strains increased plant ETR, ΦPSII, and Fv’/Fm’ measurements more than one SD greater than their respective means (Table 4). Similar to petunia plants, geranium plants treated with bacteria from the greenhouse rhizosphere collection recovered from water stress better than the negative control when comparing recovery measurements (T_3_) to measurements prior to water stress (T_1_) (Table 5).

**TABLE 4:** Influence of bacterial application on the photosynthetic health of *Pelargonium* × *hortorum* ‘Maverick Red’ plants under severe water stress (T_2_) and following recovery (T_3_).

Severe water stress (T_2_)					Recovery (T_3_)				
Strain	ETR	Fv’/Fm’	ΦPSII	Rank	Strain	ETR	Fv’/Fm’	ΦPSII	Rank
UW4 +	20.44	0.516	0.313	1	C2F7	41.63	0.745	0.636	1
C9C5	19.39	0.541	0.297	2	C4D7	41.47	0.742	0.633	2
C6C2	18.25	0.495	0.279	3	C6C2	41.45	0.739	0.633	3
C8A5	18.27	0.468	0.278	4	C9C5	40.77	0.732	0.622	4
C2F7	17.59	0.496	0.269	5	C5G2	39.61	0.725	0.604	5
C7D2	17.84	0.462	0.273	6	UW4 +	38.83	0.724	0.592	6
C5G2	17.43	0.477	0.266	7	C9C3	38.64	0.726	0.59	7
C9C3	17.06	0.487	0.261	8	C2B4	38.21	0.72	0.584	8
C1C7	17.37	0.446	0.265	9	C1C7	36.74	0.705	0.561	9
C4D7	16.85	0.465	0.258	10	C7D2	36.41	0.694	0.557	10
C2B4	16.09	0.448	0.246	11	(−) Control	35.93	0.678	0.547	11
(−) Control	12.03	0.369	0.183	12	C8A5	35.69	0.701	0.545	12
Mean	17.38	0.472	0.266		Mean	38.78	0.719	0.592	
SD	2.04	0.043	0.031		SD	2.24	0.021	0.034	

*Electron transport rate (ETR), efficiency of photosystem II (Fv’/Fm’), and quantum yield of photosystem II (ΦPSII) were measured on a single leaf of each plant per treatment (n = 6) and the mean value for each is shown. Plants treated with strains from the greenhouse rhizosphere collection were compared to the bacterial treatment control strain UW4^+^ and the uninoculated negative control. Measurements were taken 7 days after withholding water for water stress (T_2_) and 3 days after rewatering for recovery (T_3_). Means are organized based on if they are more than one standard deviation (SD) greater than the overall mean (light gray), greater than the mean but less than one SD (gray), lower than the mean but not by more than one SD (dark gray), and more than one SD lower than the mean (black). The mean values for each photosynthetic measurement were ranked from highest to lowest value, and the individual ranks were summed together to get the overall rank. Treatments with the lowest ranking sum were given the best overall rank.*

**TABLE 5:** Percent change in physiological measurements of *Pelargonium*× *hortorum* ‘Maverick Red’ after 3 days of recovery (T_3_) from being subjected to 10 days of water stress, when compared to measurements prior to water stress (T_1_).

Strain	Electrolyte leakage	ETR	Fv’/Fm’	ΦPSII
(−) Control	87.5	−18.6	−8.8	−19.0
C8A5	39.1	−18.8	−6.3	−18.9
C4D7	16.5	−4.3	−0.6	−4.2
C7D2	43.0	−14.1	−6.0	−13.8
C1C7	41.6	−11.3	−4.0	−11.0
C9C3	37.1	−10.7	−2.6	−10.5
C5G2	26.9	−7.7	−1.8	−7.6
C6C2	29.8	−2.8	0.1	−2.7
C9C5	35.5	−8.7	−2.5	−8.6
C2B4	47.2	−13.1	−3.3	−12.9
C2F7	33.7	−3.5	0.5	−3.4
UW4+	43.4	−11.7	−3.1	−11.9

*Plants treated with strains from the greenhouse rhizosphere collection were compared to the bacterial treatment control strain UW4^+^ and the uninoculated negative control.*

#### Electrolyte Leakage in Petunia and Geranium

**FIGURE 5:**
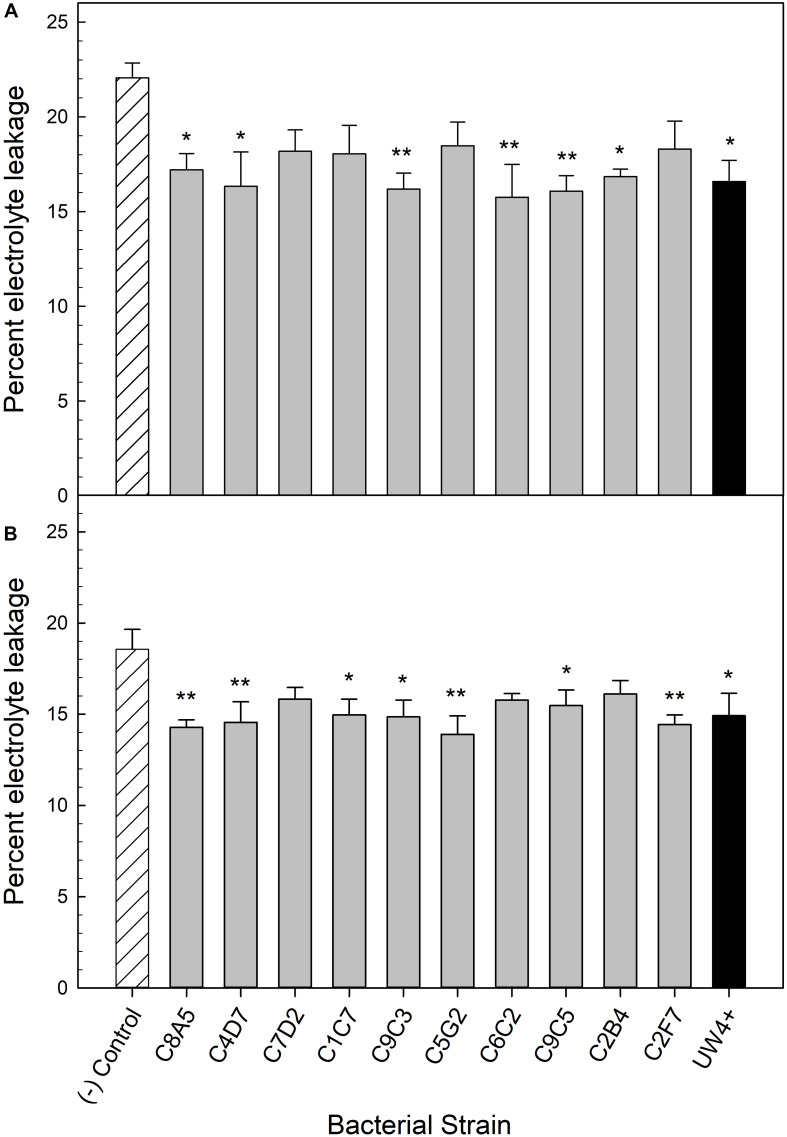
Percent electrolyte leakage of *Petunia* × *hybrida* ‘Picobella Blue’ **(A)** and *Pelargonium* × *hortorum* ‘Maverick Red’ **(B)** after 3 days of recovery from water stress (*n* = 6). Plants were treated with bacterial inoculum weekly starting at transplant and were subjected to severe water stress 5 weeks after transplant. Plants treated with strains from the greenhouse rhizosphere collection (light gray) were compared to the bacterial treatment control strain UW4^+^ (black), and the uninoculated negative control (white with lines). Bars represent mean (±SE). The asterisks indicate significance between the treatments compared to the uninoculated negative control using the Dunnett’s test; *, ** significant at *P* ≤ 0.05, and 0.01, respectively.

Application of five bacterial strains significantly decreased the percentage of electrolyte leakage in both petunia and geranium after recovery from water stress (T_3_). These included the greenhouse rhizosphere strains C8A5, C4D7, C9C3, C9C5, and the treatment control strain UW4^+^ (Figures 5A,B). Application of beneficial bacteria ameliorated the negative effects of water stress on the electrolyte leakage of petunia and geranium plants after recovering from stress (T_3_). The electrolyte leakage of negative control petunia plants increased by 54% when compared to plants before water stress, whereas plants treated with bacteria from the greenhouse rhizosphere collection and UW4^+^ had an average increase of only 11% (Table 3). The negative control geranium plants had an electrolyte leakage increase of 87% after recovery, whereas plants treated with bacteria from the greenhouse rhizosphere collection and UW4^+^ had an average increase of only 36% (Table 5) when compared to measurements pre-stress (T_1_).

## Discussion

Greenhouse ornamentals can be subjected to water deficit throughout different stages of production, shipping, and retail. There is a need to find solutions that increase plant tolerance to stress, allowing production of horticulture crops that satisfy quality demands under limited resource availability. In this study, we have established a novel collection of bacteria from the rhizosphere of greenhouse ornamentals. We then used *in vitro* and *in planta* selection methods to screen the entire greenhouse rhizosphere collection and identify strains with the ability to increase water stress tolerance in petunia and geranium. We selected petunia and geranium for this study because they are two of the most popular ornamental greenhouse crops with a combined annual wholesale value in the United States greater than $190 million (National Agricultural Statistics, 2019).

Water stress directly impacts the soil microbiome by selecting for stress-tolerant taxa. In addition, plants influence their rhizosphere microbiome during stress by altering their root exudate profile (Naylor and Coleman-Derr, 2018). We took advantage of this principle by imitating the environment that plants would go through during post-harvest water stress and selecting culturable bacteria from these plants. We isolated the general culturable bacteria from the rhizosphere as well as those able to form spores through heat treatment and selection. Sporulation allows bacteria to enter dormancy under periods of water stress, serving as an advantage over other bacteria that are less likely to persist in these environments (Hayden et al., 2012).

The application of PGPR to greenhouse ornamental crops is a useful tool to increase plant stress tolerance. The onset of water stress in greenhouse crops is rapid and can occur throughout production, making it difficult for plants to adapt to the stress and maintain superior quality. Therefore, the ability for PGPR to colonize plant roots and then stimulate plant stress responses is an attractive solution to prime ornamental crops before the onset of stress. Using previously established methods, we utilized high-throughput bioassays to identify bacteria within our collection that were able to withstand osmotic stress in PEG or produce the enzyme ACC deaminase. The use of PEG to select osmotic stress tolerant bacteria, and the ability of these bacteria to then confer water stress tolerance to plants has been well established (Forchetti et al., 2007; Vardharajula et al., 2011; Hussain et al., 2014; Asghar et al., 2015). In addition, the treatment of tomato and pepper plants with ACC-deaminase producing *Achromobacter piechaudii* stimulates plant growth during water stress, contributing to larger plant size (Mayak et al., 2004).

Many PGPR studies are conducted in greenhouses; however, these results can be difficult to relate to horticulture crop production due to the use of agronomic crop species and soil-based growing media. A previously established high-throughput greenhouse trial (Nordstedt et al., 2020) was an effective method to screen the *in vitro* selected bacteria in our collection and identify those that increased plant size or flower number of water stressed petunia plants in a greenhouse production system. In this study, the high-throughput trial was used to evaluate 80 isolates and select 10 for the validation trial with petunia and geranium.

Inhibition of shoot growth is a common plant response to water stress because it limits the leaf area available for evaporative loss. However, this decrease in plant size can lower ornamental crop quality and profitability. Application of bacteria can stimulate plant growth under severe water deficit, ameliorating the negative effects of water stress. PGPR application had the most consistent effect on water stressed petunias. Application of all ten bacterial strains significantly increased plant size. In the case of *Arthrobacter* sp. C9C5, plant size was comparable to our positive control that did not undergo any water stress (Figure 3A). These results are similar to other studies that have shown *Arthrobacter* sp. to increase the size of arabidopsis, tomato, and wheat plants grown under high saline conditions (Barriuso et al., 2008; Upadhyay and Singh, 2015; Fan et al., 2016). In addition, eight strains significantly increased plant size of water-stressed geranium (Figure 4). These eight strains consist of five different genera, including *Arthrobacter*, *Pseudarthrobacter*, *Leifsonia*, *Pseudomonas*, and *Herbaspirillum*. There is currently little evidence for *Pseudarthrobacter* spp. as a plant growth promoter other than phytoremediation (Ghasemi et al., 2018) and *in vitro* plant growth promoting properties (Mesa-Marín et al., 2019). To our knowledge, our study is the first to document a *Pseudarthrobacter* strain as a plant growth promoting bacteria under water stressed conditions. *Leifsonia* has also not been extensively studied as a PGPR, although the strain *Leifsonia xyli* SE134 has been shown to increase plant growth of cucumber, tomato, and radish under non-stressed conditions (Kang et al., 2014). *Herbaspirillum* spp. are well-documented as biological N_2_-fixer (Hayat et al., 2012). Although *Herbaspirillum seropedicae* has been shown to increase the plant size of maize under water stress (Cura et al., 2017), to our knowledge, we are the first to document *Herbaspirillum robiniae* as a PGPR under water stressed conditions. This work provides evidence of multiple bacterial species that have not been previously characterized as PGPR, adding to the diversity of PGPR that can be studied to stimulate plant growth under water stress.

Plant growth promoting rhizobacteria that can increase flower number even under water stressed conditions are of interest to the horticulture industry, and seven strains significantly increased flower number in water stressed petunia (Figure 3B). These results are similar to our previous work that identified two *Pseudomonas* spp. that increase the flower number of water-stressed petunia (Nordstedt et al., 2020). Half of the PGPR strains identified in the current study also belong to the genus *Pseudomonas* (Table 1). Many species within *Pseudomonas* have been extensively studied under a variety of conditions as plant growth promoters (Goswami et al., 2016), and our results add to the current literature by showing the species *Pseudomonas brassicacearum* and *Pseudomonas corrugata* can increase the quality of water stressed greenhouse ornamentals. Overall, this work shows that improvements in water stress tolerance allow for visual quality to be maintained longer, which can reduce plant mortality following wholesale production (Eakes et al., 1991).

Different *Pseudomonas* spp. have been shown to increase water stress tolerance in maize by reducing electrolyte leakage (Sandhya et al., 2010). This increase in membrane stability allows plants to recover more efficiently from stress, often increasing plant size (Sandhya et al., 2010; Vardharajula et al., 2011). The present study identified nine strains that decreased the electrolyte leakage in plants recovering from water stress (Figures 5A,B). Plants tended to have greater plant biomass when their electrolyte leakage was decreased after recovering from water stress (Figures 3A, 4). PSII efficiency is inversely proportional to damage in the open PSII reaction centers (Farquhar et al., 1989). Therefore, an increase in photosynthetic health via application of PGPR can delay mortality of plants. *P. brassicacearum* that delayed the decline in photosynthetic health of water stressed arabidopsis also increased the probability of plant survival (Bresson et al., 2014). In our study, we measured three different parameters of photosynthetic health: PSII efficiency (Fv’/Fm’), quantum yield of PSII (ΦPSII), and ETR. Utilizing a ranking system, we were able to select the strains with the best effect on overall photosynthetic health in our two plant species during severe water stress and recovery. The best strains were more consistent between severe water stress and recovery in petunia (Table 2). Strains C7D2, C2B4, and C1C7 were the best treatments for increasing overall photosynthetic health in petunia, while also improving plant quality of water-stressed petunias (Figures 3A,B). Noteably, these strains were originally isolated from zinnia (C7D2 and C2B4) and geranium (C1C7) rhizosphere samples. The best strains for geranium photosynthetic health were different depending on if the plants were under severe water stress or recovering from stress (Table 4). These results are consistent with Arzanesh et al. (2011) that showed different strains of *Azospirillum* had a positive influence on wheat plants that was dependent on the severity of the water stress. Strain C6C2 was identified as the best strain for increasing the overall photosynthetic health of water stressed geranium plants and also significantly increased the biomass of geranium plants after recovery from water stress. However, this strain was originally isolated from a coleus rhizosphere sample. These results, in combination with the origin of strains that increased the health of petunia, show that bacteria isolated from one plant species can have a beneficial effect on other plant species. This provides evidence of the generalist nature of these PGPR to stimulate plant growth of multiple plant species in one system, a common occurance in ornamental plant production. It is likely that environmental conditions (i.e., growing media composition, fertilizer rates, and temperature fluctuations) play a more important role than plant host in beneficial plant–microbe interactions.

We utilized *P. putida* UW4^+^ as a positive treatment control for comparison because it has been documented as an ACC deaminase producer that stimulates plant growth under abiotic stress (Grichko and Glick, 2001; Cheng et al., 2007; Farwell et al., 2007; Jodeh et al., 2015). UW4^+^ had the highest ACC deaminase activity compared to the other ten strains evaluated in the greenhouse validation trial (Figure 2). UW4^+^ also increased the quality of water stressed petunia and geranium plants when compared to the negative control, yet many of our strains performed as well or better than UW4^+^ (Figures 3AB, 4). In regard to overall photosynthetic parameters, petunia treated with UW4^+^ ranked higher than the negative control but ranked below most of our strains during water stress and recovery (Table 2). UW4^+^ had the best influence on geranium photosynthetic health during water stress and was ranked in the middle of all treatments for plants during recovery (Table 4).

Our study contributes to the current literature by establishing a large collection of diverse bacterial isolates originating from the rhizosphere of water stressed greenhouse ornamental species. In addition, we evaluated the effect of bacterial application on two economically important ornamental species, grown in a soilless peat-based media, and subjected to severe water stress. This water deficit is similar to what plants may be subjected to after production, when market-ready plants are shipped or sold at retail garden centers. Utilizing cultural inputs and inducing water stress similar to what would be experienced in greenhouse production further validates the potential for these bacteria to be used by the industry as a tool to increase plant stress tolerance and overall quality.

The commercialization of biostimulant products from isolation to formulation is timely and resource intensive (Backer et al., 2018). In addition to contributing to the scientific community, the work outlined in this study contributes significantly to the biostimulant commercialization pipeline. The methods used for isolation, screening, and validation of the PGPR strains can be used by other groups to identify beneficial strains specific to their system of interest. In conclusion, *in vitro* bioassays are effective at selecting candidate PGPR with the ability to withstand osmotic stress or produce beneficial enzymes; however, their ability to stimulate plant growth must also be validated *in planta*. PGPR can positively influence plant health and quality differently across plant species. Therefore, future research should focus on developing consortia of PGPR that can be formulated into products with broad effects across different ornamental plant species.

## Data Availability Statement

The whole-genome sequence data were deposited in the NCBI database under the BioProject no. PRJNA631210.

## Author Contributions

NN and MJ conceived the experimental design. NN collected the data, performed all analyses, and led the writing of the manuscript. MJ edited the manuscript.

## Conflict of Interest

The authors declare that the research was conducted in the absence of any commercial or financial relationships that could be construed as a potential conflict of interest.
